# Does inter-limb asymmetry matter in adolescent speed skaters?

**DOI:** 10.3389/fphys.2025.1498911

**Published:** 2025-05-15

**Authors:** Zhiyong Jin, Yufeng Wang, Binjie Zhao, Gengchao Bi, Yuzi Diao, Yao Zhang, Li Yan

**Affiliations:** ^1^ Harbin Sport University, Harbin, China; ^2^ Heilongjiang Research Institute of Sports Science, Harbin, China; ^3^ Heilongjiang Vocational College of Winter Sports, Harbin, China

**Keywords:** inter-limb difference, limb dominance, squat jump, drop jump, isokinetic testing

## Abstract

Inter-limb asymmetry (IA) has been shown to impact athletic performance, but its relationship with speed skating performance is not yet clear. To investigate the effect of IA in lower limb strength on skating time in adolescent speed skaters, 17 male adolescent speed skaters (age: 16.65 ± 0.79 years, height: 176.63 ± 6.45 cm, weight: 63.08 ± 9.51 kg) underwent body composition, isokinetic knee strength, multi-direction (vertical, horizontal and lateral) single-leg squat jump and single-leg drop jump tests (from a 20 cm box) at the end of the season to assess the IA. The results showed that most lateral single-leg squat jump (LSJ) related variables such as relative lateral peak force (7.40 ± 0.67 N/kg vs 7.03 ± 0.61 N/kg, P < 0.001, ES = 1.32), relative lateral impulse (Imp-L) (2.67 ± 0.23 Ns/kg vs 2.45 ± 0.24 N/kg, P = 0.043, ES = 0.94) and take-off velocity (2.81 ± 0.20 m/s vs 2.59 ± 0.30 m/s, P = 0.001, ES = 0.83) showed a significant left-sided dominance, and increased corresponding asymmetry prolonged 100m and 500 m skating times. Furthermore, increased asymmetry in single-leg vertical drop jump (VDJ) height also prolonged 100 m skating time. For adolescent speed skaters, the LSJ and VDJ tasks exhibit good sensitivity to the lower limb strength asymmetry, and increases in corresponding asymmetries may have negative effects on speed skating performance.

## 1 Introduction

Inter-limb asymmetry (IA) refers to the differences in morphological structure and functional performance between contralateral limbs. It is commonly quantified as the percentage difference in performance between limbs during specific tasks, which serves to describe and assess the degree of asymmetry (Bishop et al., 2018). Early applications of such concept were focused on injury prevention and risk assessment (Bishop et al., 2016). Prolonged exposure of contralateral limbs to asymmetric loads and tasks can lead to the development of IA. Consequently, IA is a widespread phenomenon in sports, and its relationship with athletic performance has received considerable attention (Maloney, 2019).

Based on the bilateral coordination control strategies, movements in sports can be categorized as bilateral symmetric (in-phase or out-of-phase) and asymmetric movements (Maloney, 2019). Previous evidence suggests that excessive IA can impair performance in symmetric movements such as sprinting and bilateral vertical jumps (VJ). Despite the inherent bilateral asymmetrical loading due to the nature of movement patterns, long-term repetitive execution of asymmetrical tasks, such as table tennis strokes, can lead to a specific increase in IA, but without negatively impacting performance. Therefore, this is considered a benign structural and functional adaptation of the body to these types of movements (Bishop et al., 2018). Furthermore, for team sports such as football and ice hockey, the specialized technical actions exhibit a mixture of bilateral symmetric and asymmetric characteristics. Repeated exposure to asymmetrical tasks, such as change of direction can lead to an increase in IA in the lower limbs of athletes, potentially affecting performance in symmetric movements like sprinting (Maloney, 2019). Therefore, athletes participating in events with multiform technical actions are more susceptible to the negative effects of IA on performance, and previous studies have primarily focused on team sports with typical related characteristics (Loturco et al., 2019; Madruga-Parera et al., 2020; Bishop et al., 2021a; Nicholson et al., 2022; Espada et al., 2023; Twible et al., 2024).

Nevertheless, unlike team sports, where athletes perform asymmetrical tasks with randomness, speed skaters (specifically refer to the long-track speed skating in the present study) must lean their bodies to the left while executing asymmetrical lower limb movements in the turns (De Koning et al., 1991). This results in significantly higher power output and blood oxygen saturation in the left leg compared to the right leg during turns (De Koning et al., 1991; Hettinga et al., 2016). Therefore, asymmetrical loading is an inherent characteristic in speed skating, long-term training may lead to an increased lower limbs asymmetry of athletes, potentially affecting the symmetric push-off movements of both legs during straight-line skating. Speed skating training is predominantly conducted on land, with land training accounting for over 50% of the total training time. Consequently, land-based specific strength training methods can also replicate the technical characteristics of ice skating in some extent, and the associated testing methods also demonstrate high ecological benefits (Stuart and Cochrane-Snyman, 2022). The horizontal and lateral single-leg squat jump (SLSJ) has been shown to closely resemble the neuromuscular patterns of the skating push-off movements, and the peak take-off velocity is highly correlated with the results (Zukowski et al., 2023b). However, it has been shown that there are no significant differences in peak velocity between legs in the horizontal and lateral single-leg jump for speed skaters. Given that the outcome measure of take-off velocity in the study may have relatively poor sensitivity to the IA index (Virgile and Bishop, 2021), the results should be interpreted with caution. Alongside SJ, drop jump (DJ) are also widely incorporated into the strength training programs of speed skating, as the speed skating starting technique shares similarities with on-land starting techniques, where both require rapid stretch-shortening cycle (SSC) contractions of the lower limbs and demand a high level of reactive strength (Song et al., 2017). Despite the fact that DJ performance can evaluate or train the SSC capacity of the lower limbs, and it exhibits directionality, such as horizontal DJ performance being superior in predicting sprinting performance compared to vertical direction (Schuster and Jones, 2016). According to the author’s limited knowledge, there is currently no study analyzing the impact of the DJ performance on speed skating performance, and the related IA characteristics remains unknown.

Long-term specialized training has been shown to induce specific changes in brain tissue, which can affect limb coordination strategies and neuromuscular function (Nakata et al., 2010). Therefore, it is necessary to clarify the relationship between IA and athletic performance during adolescence and implement timely interventions, as researchers have done across various sports (Pontaga and Zidens, 2014; Pardos-Mainer et al., 2020; Blagrove et al., 2021; Pamuk et al., 2023). Neuro-plasticity have been observed in speed skaters (Park et al., 2013), however, reports of lower limb strength performance asymmetry among adolescent speed skaters is scarce, and the relationship between it and results remains unclear.

Considering that IA is highly task-specific, there are differences in the same metric across different tasks and between different metrics within the same task. Therefore, IA assessment should be based on tasks that closely resemble the technical action and neuromuscular patterns of the sport-specific technique (Bishop et al., 2018). However, previous studies comparing the correlation between land tests and on-ice performance have primarily focused on adult athletes (Zhang and Xia, 2011; Zukowski et al., 2023b), it remains unclear how effective land tests are in predicting on-ice performance in adolescent athletes. Therefore, the objectives of this study include: (1) assessing the characteristics of bilateral lower limb strength performance in adolescent speed skaters; and (2) examining the impact of critical strength performance variables asymmetries that are significantly related to the results on skating time. We hypothesize that the left lower limb strength performance is superior to the right side, and increases in critical variables asymmetries are associated with prolonged skating time.

## 2 Materials and methods

### 2.1 Subjects

17 professional male adolescent speed skaters from the Heilongjiang Ice Training Center voluntarily participated in the study (age: 16.65 ± 0.79 years, height: 176.63 ± 6.45 cm, weight: 63.08 ± 9.51 kg), who participated in the National Junior Speed Skating Championships or National U17 Speed Skating League. All participants had maintained regular training over the past year and had at least 3 years of prior training experience, with no athletic injuries occurred in the past 6 months and were in good health during the testing period. This study adhered to the Declaration of Helsinki and was approved by the institutional ethics committee (code: 2024013). Informed consent was obtained from all participants and their guardians prior to the experiment.

### 2.2 Procedures

**FIGURE 1:**
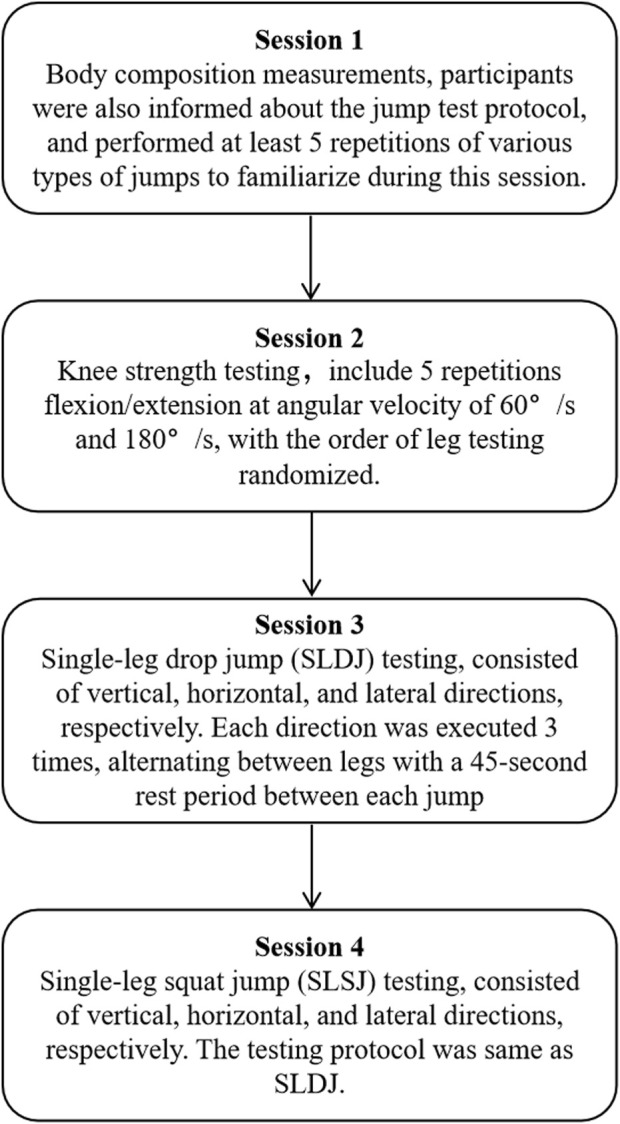
Testing procedures.

Testing was consisted of four sessions (Figure 1), and conducted during the speed skating competition season (February to March). Participants were instructed to abstain from alcohol and caffeine intake 3 hours prior to each test session. Each test session was separated by at least 48 h, and no high-intensity training was performed on the day before strength session. The first session included the body composition measurements. In addition, the participants were informed about the jump test protocol and performed at least five repetitions of various types of jumps to familiarize. During the second session, isokinetic knee strength was measured. In the third and fourth sessions, single-leg drop jumps (SLDJ) were performed, and SLSJ were measured in the vertical, horizontal, and lateral directions. In the fourth session (SLSJ), three participants withdrew due to personal reasons, resulting in a sample size of n = 14 for the fourth session. Prior to the isokinetic and jump testing, a standardized warm-up was conducted, which included a 5-min cycling bout at 60 W, followed by 3 min of dynamic stretching, and 3 min of familiarization with the testing procedures. To minimize the influence of circadian rhythms, all testing was conducted during the same time slot each day (8:00 a.m. to 11:00 a.m.) (Pradhan et al., 2024). The best performance from the most recent two public competitions for each athlete was selected for correlation analysis, with results reported to two decimal places. The skating time data were obtained from the official website of the Chinese Skating Association (https://www.chinacsa.net.cn). All competitions were held between January and March, a period during which athletes generally achieve their peak athletic levels (McCarthy, 2003), and the altitudes of the competition venues were similar (140–200 m).

#### 2.2.1 Body composition measurements

The InBody770 bioelectrical impedance analyzer (InBody Co., Seoul, South Korea) was used to measure the body fat, and muscle mass of participants. This method has been shown to have high retest reliability and validity (*r* ^2^ = 0.96; CV = 0.15%; ICC = 0.96) and can serve as an effective alternative to dual-energy X-ray absorptiometry (DXA) for measuring body composition (McLester et al., 2020; Lee et al., 2021).

#### 2.2.2 Isokinetic knee strength testing

**FIGURE 2:**
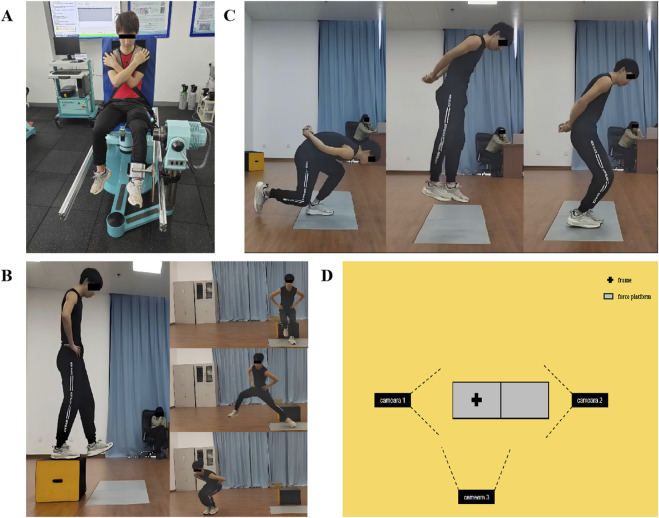
Diagrams of the Isokinetic knee strength testing **(A)**, Single-leg drop jump **(B)**, Single-leg squat jump **(C)**, Plan of the jump testing site **(D)**.

The isokinetic knee strength testing was conducted using the Con-Trex MJ isokinetic dynamometer (Con-Trex AG, Dubendorf, Switzerland). The reliability of this instrument has been previously validated (Maffiuletti et al., 2007). The backrest of the seat was adjusted to 85°, participants crossed their arms over chests, and the trunk was secured to the backrest with straps. The rotational axis of the dynamometer was aligned with the lateral epicondyle of the tested knee, and the distal end of the rotational arm was attached approximately 2–3 cm above the ankle joint. The tested thigh was strapped with a magic tape for stabilization (Figure 2A). The range of motion for the knee was set to 0°–80°, and gravitational corrections were applied prior to testing. Prior to the formal testing, participants performed five sub-maximal knee flexion-extension movements for warm-up. The testing protocol was conducted at angular velocity of 60°/s and 180°/s, with the order of leg testing randomized. A total of four sets of tests were conducted, each set consisted of five repetitions of knee flexion-extension, with a 2-min rest period between sets. Verbal encouragement was provided to the participants throughout the testing.

#### 2.2.3 Single-leg drop jump

Kinetic data during the take-off phase were collected using a Kistler 3-D force platform (Kistler Instrument AG, Winterthur, Switzerland) at a sampling frequency of 1,000 Hz. Three cameras (SONY HDR-FX1000E, Tokyo, Japan) were positioned 5 m in front of, to the left of, and to the right of the participant to capture the take-off action at a frame rate of 50 Hz for kinematic analysis. The principal axes of the left and right cameras were oriented perpendicular to the sagittal plane of participants, while the principal axis of the front camera was oriented perpendicular to the frontal plane of participants. A PEAK (3 m × 3 m × 3 m) radial calibration frame (composed of 24 points) was used to calibrate the 3-D motion space around the force platform (Figure 2D). Participants stood on a 20 cm high jump box (20 cm away from the force platform) with their hands placed on their hips (Holm et al., 2008). Upon the experimenter’s command, participants stepped forward with the tested limb, landing naturally on the force platform and then performing a rapid jump in the vertical, forward, and lateral directions. Throughout the test, participants kept hands on the hips, and landed with both feet (with the feet touching down sequentially in the lateral direction) (Figure 2B). Prior to testing, participants were instructed to jump as high or as far as possible while minimizing the take-off time. Each direction of the SLDJ task was executed 3 times, alternating between legs with a 45-s rest period between each jump (Dobbs et al., 2015). After completing all jumps in the same direction (6 jumps in total), changed the direction with a 2-min rest period between directional changes. The order of directions was randomized. Participants were required to repeat the test if any of the following occurred: jumping rather than naturally falling onto the force platform; not keeping hands on the hips throughout the take-off; failing to maintain balance after landing.

#### 2.2.4 Single-leg squat jump

The kinetic and kinematic data collecting method was same as the SLDJ test. Participants stood on one leg on the force platform, with the supporting knee flexed to approximately 90°, and another leg bent the knee with the foot tip off the ground. Meanwhile, participants leaned forward and kept hands behind their back, simulating the starting position of skating push-off. Participants held this position for 2–3 s before receiving the command to perform a jump in the vertical, horizontal, and lateral directions. Throughout the test, participants kept hands behind their back, and landed with both feet (with the feet touching down sequentially in the lateral direction) (Figure 2C). The testing sequence and rest intervals were same as SLDJ. Participants were required to repeat the test if any of the following occurred: non-supporting leg touched the ground before take-off; not keeping hands behind their back throughout the take-off; failing to maintain balance after landing.

### 2.3 Data processing

**FIGURE 3:**
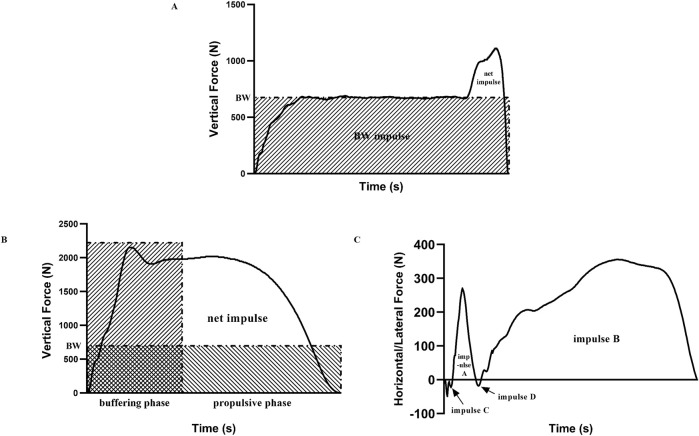
Schematic diagram of the vertical net impulse during the SLSJ take-off phase **(A)**, BW = body weight, the net impulse is calculated as the total impulse minus the BW impulse. Vertical net impulse during the SLDJ propulsive phase **(B)**, the vertical net impulse was calculated as the total vertical impulse during the propulsive phase minus the BW impulse during the same phase. Horizontal/lateral net impulse during the SLDJ propulsive phase **(C)**, the horizontal/lateral net impulse is calculated as the sum of impulse A and impulse B minus the sum of impulse C and impulse D

The video files were imported into SIMI Motion 8.0 (SIMI Reality Motion Systems, Germany) for kinematic analysis. The raw data were smoothed using a low-pass digital filter, residual analysis indicated a cut-off frequency of 6–10 Hz (Winter 2009). In the SLSJ, manual digitization was performed to mark the foot tip, heel, medial aspect of the first metatarsal, and lateral aspect of the fifth metatarsal. The horizontal SLSJ jump distance was defined as the distance from the foot tip of the support leg at take-off to the heel of the posterior foot after landing. The lateral SLSJ jump distance was defined as the distance from the medial aspect of the first metatarsal of the support leg at take-off to the lateral aspect of the fifth metatarsal of the same leg after landing. Both horizontal and lateral jump distances were normalized by height (Holm et al., 2008), and the vertical jumping height was calculated from the force-time curve. In the SLDJ, 21 anatomical points were manually digitalized for analyzing, including the head, bilateral hands, elbows, shoulders, hips, knees, ankles, toes, heels, medial aspect of the first metatarsal, and lateral aspect of the fifth metatarsals. The body center of gravity (BCoG) were calculated based on the Hanavan human mechanics model (Hanavan, 1964). The jumping distances and heights in the SLDJ were calculated and processed similarly to the SLSJ, and the velocity of the BCoG at the first touch-down moment was also recorded. The event synchronization technique (take-off moment) was applied to achieve multi-camera synchronization through the SIMI Motion Twin. The Direct Linear Transformation (DLT) algorithm was used to establish the three-dimensional coordinates, and the intra-class correlation coefficient (ICC) of 0.97 for the BCoG velocity indicates reliable manual digitization (Atkinson and Nevill, 1998). The kinetic data were imported into MATLAB for analysis, and smoothed at 75 Hz. The vertical force exceeding 10 N above the body weight (BW) were defined as the start of the SLSJ, and values below 25 N were defined as the moment of take-off (Hunter et al., 2005). In the SLSJ, the impulse and peak forces (PF) in the X (horizontal), Y (lateral), and Z (vertical) directions were calculated and recorded from the force-time curve, along with the rate of force development (RFD) and take-off velocity. For better comprehension, the vertical take-off velocity was converted to height. The horizontal and lateral impulse were calculated as the area under the force-time curve (Figure 3C), while the net impulse was calculated in the vertical direction according to Hunter et al. (2005) (Figures 3A, B), the PF and impulse were normalized by BW. The RFD (Equation 1), take-off velocity (Equations 2–4), and jump height (Equation 5) were calculated according to the methods presented by Ebben et al. (2010) and Guo et al. (2020):

$$RFD=\frac{F_{peak}-F_{initial}}{∆T_{peak}}$$ (1)

F_peak_ = Peak GRF, F_initial_ = Initial GRF on landing, ∆ T_peak_ = Time from the initiation to the peak GRF.

$$V_{take-off}=\sqrt{{V_{v}}^{2}+{V_{h/l}}^{2}}$$ (2)

$$V_{v}=\int_{0}^{T_{o}}\left(F_{v}/m-g\right)dt$$ (3)

$$V_{h/l}=\int_{0}^{T_{o}}\left(F_{h/l}/m\right)dt$$ (4)

$$H={V_{v}}^{2}/2g={\left(\int_{0}^{T_{o}}\left(F_{v}/m-g\right)dt\right)}^{2}/2g$$ (5)

V_take-_off = take-off velocity, V_v_ = vertical take-off velocity, V_h/L_ = horizontal/lateral take-off velocity, T_o_ = take-off time, F_v_ = vertical GRF, m = body mass, g = gravitational acceleration, F_h/L_ = horizontal/lateral GRF, H = vertical jump height.

In the SLDJ, the change in BCoG velocity (including the take-off velocity) was calculated based on the method of McMahon et al. (2021) by combining the first touch-down BCoG velocity (Equations 6–8). Similarly, the vertical SLDJ take-off velocity was converted to jump height using the same method as for the vertical SLSJ. The formula for calculating BCOG velocity is as follows:

$$V_{rb}=\sqrt{{V_{vb}}^{2}+{V_{hb/lb}}^{2}}$$ (6)

$$V_{vb}=\left(\int_{0}^{T_{o}}\left(F_{v}/m-g\right)dt\right)-V_{vt}$$ (7)

$$V_{hb/lb}=\left(\int_{0}^{T_{o}}\left(F_{h/l}/m\right)dt\right)+V_{ht/lt}$$ (8)

V_r_ = resultant velocity of BCOG, V_vb_ = vertical velocity of BCOG, V_hb/lb_ = horizontal/lateral velocity of BCOG, T_o_ = take-off time, F_v_ = vertical GRF, m = body mass, g = gravitational acceleration, V_vt_ = vertical touch-down velocity of BCOG, F_h/l_ = horizontal/lateral GRF, V_ht/lt_ = horizontal/lateral touch-down velocity of BCOG.

The moment when the BCOG velocity reached 0 m/s was defined as the instant of transition from eccentric to concentric action of the support leg, the period from this moment to the take-off was defined as the propulsive phase. The net impulse and PF in the X, Y, and Z directions during the propulsive phase were calculated and recorded (Figures 3B,C), also normalized by BW. Additionally, considering the time as a critical performance variable affecting the SSC, we normalized variables in the SLDJ using the reactive strength index (RSI) based on the take-off time (Holm et al., 2008).

### 2.4 Statistical analysis

SPSS 21.0 (IBM, Armonk, NY) was used for statistical analysis, results are presented as mean ± standard deviation (SD). For the SLSJ and SLDJ tests, following González-García et al. (2024) recommended, the first jump was excluded, and the mean values of the relevant variables from the subsequent two jumps were used for analysis. The normality of data distribution was evaluated using the Shapiro-Wilk test. For normally distributed data, Pearson correlation analysis and paired samples t-tests were used to examine the relationships between dominance side variables, IA and the results, and the inter-limb differences for variables. For non-normally distributed data, Spearman rank correlation analysis and Wilcoxon signed-rank tests were used for the aforementioned analyses. In addition, quartile splits were performed for IA variables without significant correlations with the results, and independent samples t-tests or Mann-Whitney U tests were used to compare the skating time differences between the lowest quartile (Q1) and highest quartile (Q4) asymmetry group of IA. Cohen’s d effect size was used to assess the magnitude of inter-limb or group differences, where: ES < 0.20 = trivial, 0.20–0.49 = small, 0.50–0.79 = medium, and ≥0.8 = large. And the correlations magnitude were defined according to Hopkins (2002): r = 0–0.1 (trivial), 0.1–0.3 (small), 0.31–0.5 (moderate), 0.51–0.7 (large), 0.71–0.9 (extremely large) and 0.91–1.0 (almost perfect). For the SLSJ and SLDJ tests, within-session reliability of test measures were quantified using a two-way random intra-class correlation coefficient (ICC) with absolute agreement inclusive of 95% confidence intervals (CI) and the coefficient of variation (CV). Interpretation of the reliability followed the recommendations of Bradshaw et al. (2010): ICC >0.67 and CV < 10% = good, ICC <0.67 or CV > 10% = moderate, ICC <0.67 and CV > 10% = poor. The IA was quantified using the formula from Bishop et al. (2021b) (100 - (100/maximum value × minimum value)) to express the degree of asymmetry as a percentage. An IF function was added to indicate the direction of asymmetry (left < right, −1, 1), with positive values indicating left-sided dominance and negative values indicating right-sided dominance. In addition, the Kappa coefficient was calculated to evaluate the consistency of asymmetry direction across tests. Consistency levels were interpreted according to Viera and Garrett (2005) as follows, where Kappa values: 0.01–0.20 = slight, 0.21–0.40 = fair, 0.41–0.60 = moderate, 0.61–0.80 = substantial, 0.81–0.99 = almost perfect. Furthermore, given the scarcity of relevant previous studies, a *post hoc* power analysis was conducted (G*Power v. 3.1.9.7) based on the sample size in this study, the results indicated an average statistical power of 0.864 for the relevant variables (alpha level set at 0.05).

## 3 Results

### 3.1 Feature of IA

**FIGURE 4:**
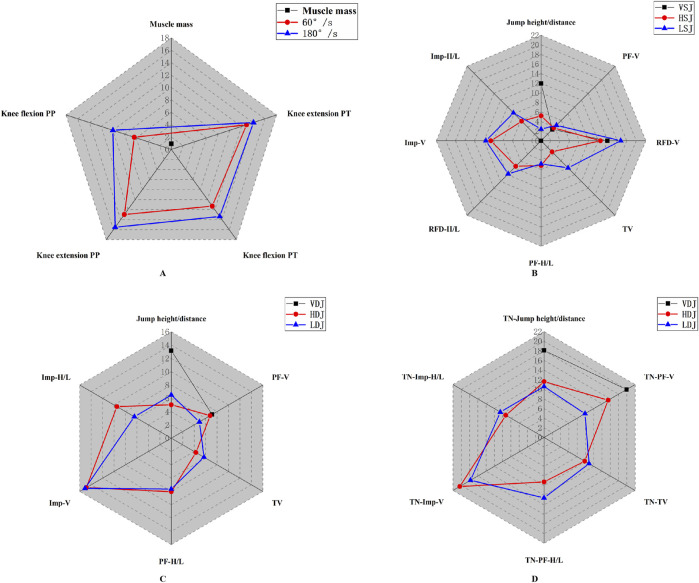
Radar chart depicting asymmetry in isokinetic knee strength and muscle mass **(A)**, SLSJ **(B)**, SLDJ **(C)**, TN-SLDJ **(D)**. V, vertical; H, horizontal; L, lateral; PP, peak power; PT, peak torque; Imp, impulse; PF, peak force; TV, take-off velocity; RFD, rate of force development; TN, time normalized.

The general characteristics of all tests can be found in Supplementary Tables S1–S4. Except for vertical net impulse, the retest reliability of other variables was moderate to good. In isokinetic testing, significant right-side dominance was observed in relative knee extension PT (t = −3.403, P = 0.004, effect size ES = 1.17), PP (t = −4.310, P = 0.001, ES = 0.94) at 60°/s, and PP (t = −2.202, P = 0.043, ES = 0.76) at 180°/s. However, in the lateral single-leg squat jump (LSJ) test, most variables exhibited significant left-side dominance, including relative vertical peak force (PF-V) (t = 2.537, P = 0.025, ES = 0.47), lateral peak force (PF-L) (t = 4.863, P < 0.001, ES = 1.32), lateral impulse (Imp-L) (Z = −2.023, P = 0.043, ES = 0.94), and take-off velocity (TV) (Z = −3.296, P = 0.001, ES = 0.83). Limb dominance (Kappa coefficients = −0.29–0.65) (Supplementary Tables S5–S8; Supplementary Figure S1) and asymmetry magnitude (0.92%–20.35%) (Figure 4; Supplementary Tables S9 and S10) exhibited variability across tests.

### 3.2 Relationship between body composition, strength performance and skating time

Table 1 shows that the absolute lower limb muscle mass has stronger correlations with 100 m and 500 m skating time (*r* = −0.63, P = 0.007; *r* = −0.69, P = 0.002) than the relative values (*r* = −0.53, P = 0.024; *r* = −0.52, P = 0.026), and the knee flexion PP at 180°/s is largely correlated with skating time (*r* = −0.60, P = 0.012; *r* = −0.58, P = 0.015). In terms of jumping performance (Table 2), the correlations between lateral jump-related variables and skating time (*r* = −0.63 to −0.77) were higher than those for horizontal direction (*r* = −0.54 to −0.60) in the SLSJ, with TV showing the highest correlation. Similarly, variables significantly correlated with performance in the SLDJ were mostly concentrated in lateral jump. However, in all three directions, vertical performance variables such as the jump height and PF-V were consistently correlated with skating time (*r* = −0.50 to −0.55), but decreased after time normalization.

**TABLE 1:** Correlation between body composition, isokinetic knee strength, and skating time.

Variables (n = 17)	100 m	500 m
Body composition		
Body fat	0.17	0.07
Absolute lower limb muscle mass	−0.63**	−0.69**
Relative lower limb muscle mass	−0.53*	−0.52*
Isokinetic test		
Relative knee extension PT at 60°/s	−0.01	−0.15
Relative knee flexion PT at 60°/s	−0.08	−0.19
Relative knee extension PT at 180°/s	−0.11	−0.12
Relative knee flexion PT at 180°/s	−0.09	−0.09
Relative knee extension PP at 60°/s	−0.01	−0.13
Relative knee flexion PP at 60°/s	−0.42	−0.47
Relative knee extension PP at 180°/s	−0.15	−0.14
Relative knee flexion PP at 180°/s	−0.60*	−0.58*

Abbreviations: PT, peak force; PP, peak power.

**TABLE 2:** Correlation between jump tests and skating time.

SLSJ (n = 14)	100 m	500 m	SLDJ (n = 17)	100 m	500 m	TN-SLDJ (n = 17)	100 m	500 m
**Vertical**			**Vertical**			**Vertical**		
Jump height	−0.52	−0.46	Jump height	−0.53*	−0.30	Jump height	−0.50*	−0.35
PF-V	0.08	0.06	PF-V	−0.28	−0.18	PF-V	0.16	0.18
RFD-V	−0.04	−0.12						
**Horizontal**			**Horizontal**			**Horizontal**		
Jump distance	−0.56*	−0.54*	Jump distance	−0.46	−0.31	Jump distance	−0.35	−0.34
TV	−0.60*	−0.54*	TV	−0.38	−0.22	TV	−0.42	−0.42
PF-V	0.24	0.18	PF-V	−0.55*	−0.54*	PF-V	−0.39	−0.42
RFD-V	−0.07	−0.14	PF-H	−0.25	−0.26	PF-H	−0.27	−0.31
PF-H	−0.42	−0.43	Imp-V	−0.44	−0.34	Imp-V	−0.39	−0.30
RFD-H	−0.34	−0.37	Imp-H	−0.34	−0.33	Imp-H	−0.32	−0.22
Imp-V	0.06	0.01						
Imp-H	−0.55*	−0.54*						
**Lateral**			**Lateral**			**Lateral**		
Jump distance	−0.41	−0.35	Jump distance	−0.38	−0.43	Jump distance	−0.37	−0.37
TV	−0.77**	−0.76**	TV	−0.56*	−0.48	TV	−0.39	−0.27
PF-V	−0.32	−0.45	PF-V	−0.53*	−0.50*	PF-V	−0.42	−0.40
RFD-V	−0.22	−0.28	PF-L	−0.55*	−0.54*	PF-L	−0.54*	−0.45
PF-L	−0.73**	−0.65*	Imp-V	−0.34	−0.34	Imp-V	−0.44	−0.30
RFD-L	−0.63*	−0.64*	Imp-L	−0.32	−0.33	Imp-L	−0.39	−0.22
Imp-V	−0.32	−0.30						
Imp-L	−0.63*	−0.65*						

Abbreviations: SLSJ, single-leg squat jump; SLDJ, single-leg drop jump; TN, time-normalized; PF, peak force; RFD, rate of force development; TV, take-off velocity; Imp, impulse.

*, P < 0.05; **, P < 0.01.

### 3.3 Relationship between critical variables asymmetries and skating time

**FIGURE 5:**
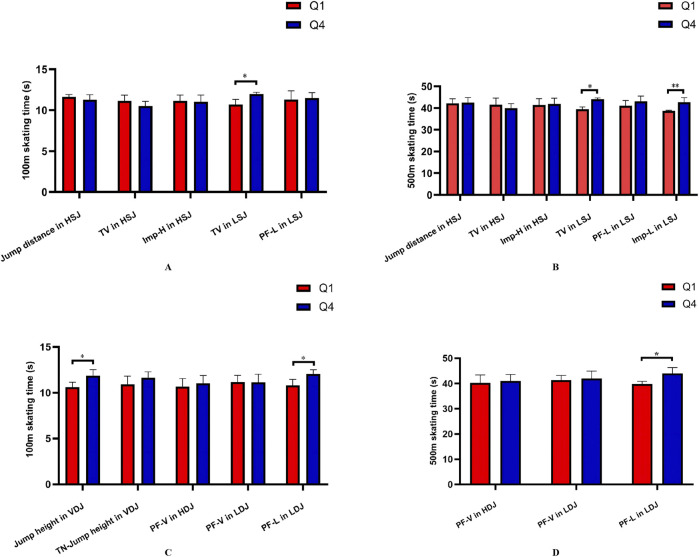
Comparison of 100 m **(A)**, 500 m **(B)** skating time between asymmetry groups (SLSJ). 100 m **(C)**, 500 m **(D)** skating time between asymmetry groups (SLDJ). Q1, lowest quartile group of asymmetry; Q4, highest quartile group of asymmetry; V, vertical; H, horizontal; L, lateral; HSJ, horizontal single-leg squat jump; LSJ, lateral single-leg squat jump; VDJ, vertical single-leg drop jump; HDJ, horizontal single-leg drop jump; LDJ, lateral single-leg drop jump; TV, take-off velocity; Imp, impulse; PF, peak force; *, P < 0.05; **, P < 0.01; ns, no significant difference.

The relationship between the IA of critical variables and skating time was examined. The results indicate that the lower limb muscle mass and isokinetic knee strength asymmetries were not associated with skating time. Furthermore, in the jumping tests (Table 3), regardless of SLSJ or SLDJ, only the lateral jump-related variables asymmetries and skating time exhibited a significant correlation (*r* = −0.54 to −0.57). To further investigate the correlation between the critical variables asymmetries and skating time, a quartile division was applied for critical variables asymmetries not significantly correlated with skating time, and the differences in skating time between the lowest quartile (Q1) and highest quartile (Q4) asymmetry groups were compared (Figure 5). Significant differences in skating time were observed between groups for the following variables: TV (100 m: t = −3.324, P = 0.029, ES = 2.71; 500 m: t = −6.476, P = 0.003, ES = 5.29), Imp-L in LSJ (500 m: t = −3.159, P = 0.034, ES = 2.58), jump height in VDJ (100 m: t = −3.071, P = 0.022, ES = 2.17), and PF-L in LDJ (100 m: t = −3.306, P = 0.016, ES = 2.34; 500 m: t = −3.258, P = 0.017, ES = 2.30).

**TABLE 3:** Correlation between the critical variables asymmetries in jumping tests and skating time.

SLSJ (n = 14)	100 m	500 m	SLDJ (n = 17)	100 m	500 m	TN-SLDJ (n = 17)	100 m	500 m
**Horizontal**			**Vertical**			**Vertical**		
Jump distance	−0.01	0.23	Jump height	0.38		Jump height	0.25	
TV	−0.50	−0.32	**Horizontal**			**Lateral**		
Imp-H	−0.23	−0.05	PF-V	0.12	0.17	PF-L	0.56*	
**Lateral**			**Lateral**					
TV	0.50	0.47	TV	0.57*	0.55*			
PF-L	0.16	0.42	PF-V	0.20	0.31			
RFD-L	0.57*	0.54*	PF-L	0.44	0.43			
Imp-L	0.56*	−0.47						

Abbreviations: SLSJ, single-leg squat jump; SLDJ, single-leg drop jump; TN, time-normalized; PF, peak force; RFD, rate of force development; TV, take-off velocity; Imp, impulse.

*, P < 0.05.

## 4 Discussion

The purpose of this study was to evaluate the bilateral lower limb strength characteristics in adolescent speed skaters, and to investigate the relationship between critical variables asymmetries and skating time. The results partially support the hypothesis. Although there were differences in bilateral lower limb strength performance, only the TV in the HSJ and most LSJ related performance variables showed a significant left-side dominance. Limb dominance did not consistently favors the same side, and there were considerable differences in the asymmetry magnitude across tests. Furthermore, a positive relationship was observed between critical variables asymmetries and skating time.

The asymmetrical stroking technique in speed skating on corners can lead to increased blood flow restriction (BFR) in the right leg, with a significant decrease in oxygen saturation compared to the left leg (Born et al., 2014). BFR has been shown to promote muscle hypertrophy through increased secretion of growth hormone and activation of satellite cells (Hwang and Willoughby, 2019). However, no significant difference in bilateral lower limb muscle mass was observed in the subjects. Hettinga et al. (2016) showed that although speed skaters experience a significant decrease in oxygen saturation in the right leg during the corner phase, rapid re-oxygenation occurs during straight-line skating, thereby alleviating the BFR in the right leg. Consequently, the difference in muscle hypertrophy effects caused by BFR are diminished, leading to relatively balanced muscle mass in both legs (0.92% ± 0.64%), and the low degree of muscle mass asymmetry may also not be sufficient to effectively distinguish speed skaters of different competitive levels. It should be noted that the participants in this study were adolescent athletes, considering the typical asymmetric loading characteristics of speed skating, the lower limb muscle mass asymmetry may increase with training years, as well as the natural development (Luk et al., 2014). Future studies could evaluate adult athletes to further investigate the relationship between lower limb muscle mass asymmetry and speed skating performance. Furthermore, unlike most speed sports (Knechtle et al., 2010; Tsukahara et al., 2020), although increased relative muscle mass of the lower limb was associated with faster skating times (r = −0.52 to −0.53), its correlation was weaker compared to absolute values (r = −0.63 to −0.69), and no association was observed between body fat percentage and skating times. Mu and Xia (2013) reported the same result in adult speed skating athletes, which may be related to the speed skating specific technique that relies less on overcoming self-gravity (Konings et al., 2015).

Isokinetic strength testing revealed a laterality in the knee strength of speed skaters, the right leg exhibited higher relative knee extension PT, PP at 60°/s, and PP at 180°/s compared to the left leg (P < 0.05), consistent with the findings of Xia (2011). De Koning et al. (1991) demonstrated that speed skaters exhibit differences in the coordination patterns of bilateral lower limb during push-off, the net torque and average power at the right knee joint are significantly higher than those on the left side, with the push-off impetus primarily driven by knee extension in right stroke. Given that isokinetic knee extension strength testing primarily reflect the single-joint strength of the quadriceps, the corresponding right-side dominance may be attributed to this. Nonetheless, only the relative knee flexion PP at 180°/s was significantly correlated with skating time, while no correlation was found between the corresponding asymmetry and skating time. Indicating that due to the constraints of single-joint isolated movements, isokinetic knee strength testing may not effectively reflect the specific strength of speed skaters, consequently the corresponding asymmetry was not related to skating time. Nevertheless, considering the evidence of relationship between higher asymmetry in isokinetic knee strength and increased knee injury risk (Eagle et al., 2019), practitioners can still regularly monitor the corresponding asymmetry to prevent potential injuries.

In contrast to the right-side dominance in isokinetic knee extension strength, a significant left-side dominance in TV (P < 0.05) was observed in the LSJ and HSJ tests. According to the study by De Koning et al. (1991), the left leg exhibits higher muscle activation and approximately 30% greater output power compared to the right leg in corner stroke, the long-term repetition of the asymmetrical stroking movement pattern in corner may be the primary cause of the aforementioned phenomenon. Nevertheless, Zukowski et al. (2023a) did not report bilateral TV differences in HSJ and LSJ in speed skaters. In their study, participants were not restricted in arm swing, which may have affected the test outcomes by coordination strategies (Guo et al., 2020). When detecting the performance variables derived from force-time curves, significant left-side dominance was observed only in LSJ for variables such as PF-L and Imp-L. Nevertheless, the magnitude of impulse asymmetry is greater than that previously observed in ice hockey (Twible et al., 2024), another ice-based sport. This discrepancy may be associated with the consistent counterclockwise skating direction of speed skaters, which results in a constant asymmetric loading.

It is noteworthy that, despite most performance variables exhibited the left-side dominance in lateral jump tasks, still observed a significantly higher RFD in the right side during the LSJ task. This phenomenon may be related to the right leg reaching PP earlier in the curve skating (De Koning et al., 1991), and it also suggests that the asymmetry direction can vary among variables even within the same task. Therefore, when assessing the directional characteristics of IA, emphasis should be placed not only on the differences across different tasks, but also on the differences among different variables within the same task, giving different consideration to different variables even within the same task is of critical importance. For example, differently from other variables, RFD is the most sensitive variable to conditioning and deconditioning (Ruggiero and Gruber, 2024), which may result in a different asymmetry direction.

Furthermore, the aforementioned performance variables in LSJ were also significantly correlated with skating time, combined with the correlation between TV in LSJ (*r* = −0.76 to −0.77) and skating times was higher than that between TV in HSJ (*r* = −0.54 to −0.60) and skating times, indicating that the LSJ task may be more effective than the HSJ in detecting IA in speed skaters. Different to the terrestrial locomotion, where the propulsive force is primarily provided by the GRF in the same direction as movement, speed skating primarily relies on lateral pushing force to move forward, which act as the normal force in the direction of motion (Houdijk et al., 2002). Therefore, the LSJ may have higher fidelity in simulating the skating push-off action compared to the HSJ. Just as Afonso et al. emphasized that due to the task-specific nature of IA, there are substantial differences in IA across different tasks. Therefore, when analyzing the relationship between IA and athletic performance, IA assessments should be based on tasks that are similar to the specialized technical neuromuscular control and coordination strategies (Afonso et al., 2022). It is recommended to prioritize the LSJ task when assessing IA in speed skaters with SLSJ tests.

In further comparing the associations between critical variables in SLSJ and skating time, only LSJ related performance variables such as RFD-L and Imp-L were significantly correlated with skating times (*r* = 0.54–0.57). Due to the minimal friction between the blades and the ice in the horizontal direction, speed skaters primarily rely on alternating lateral push-off to move forward, and the normal reaction force which is perpendicular to the gliding direction directly affects the effectiveness of push-off (Konings et al., 2015). Consequently, a high level of the RFD-L indicates that athletes can reach PF earlier and shorten the push-off time (Semmler, 2002), thereby increasing power output per unit time. And the Imp-L is the integral of the lateral force over the push-off time, an increase in the Imp-L can enhance the total push-off power output (Kozinc, 2022). Theoretically, reducing IA by enhancing the non-dominant side performance without compromising the dominant side performance, could increase the overall bilateral push-off power output. In addition, due to the alternating push-off with both legs, a larger asymmetry might cause a certain lateral deviation in the skating trajectory, potentially leading to an extended total skating distance (Kamst et al., 2012). Therefore, over-development of LSJ performance asymmetry might impair the skating performance. Notably, although PF-L asymmetry was not associated with skating time, impulse asymmetry can be better improved by increasing RFD on the non-dominant side along with enhancing PF. Furthermore, PF has been identified as a critical performance variable in similar roller skating events (Aedo-Muñoz et al., 2022), indicates that improvements in PF-L asymmetry may also benefit speed skaters. Furthermore, while no correlation was observed between the LSJ TV asymmetry and skating time, quartile statistical analyses demonstrated that athletes with lower TV asymmetry in LSJ had faster skating times, indicating that the LSJ TV asymmetry can still distinguish different levels of speed skating competitive ability to a certain extent. Nevertheless, aforementioned IA variables significantly correlated with skating time need to be obtained through the force platform, which may not be conducive to routine monitoring due to the ecological benefits. In theory, jump distance is primarily influenced by take-off angle and velocity, with the former varying within a relatively narrow range, making take-off velocity the main factor affecting jump distance (Graham-Smith and Lees, 2005). However, no significant correlation was observed between LSJ jump distance and its corresponding asymmetry with skating time, which may be related to the subjects landing on both feet sequentially, with lower limb flexibility affected the results (Cerrillo-Sanchis et al., 2024). Future studies may employ a simultaneous double-foot landing protocol to further investigate the impact of LSJ jump distance and corresponding asymmetry on skating performance, thereby improving potential practicality for routine monitoring.

Asymmetries in SLDJ performance variables were also found to be associated with skating time, the group with high VDJ jump height asymmetry demonstrated significantly longer 100 m skating time compared to the group with low asymmetry, consistent with the findings by Bishop et al. (2022) in soccer, where an increase in VDJ jump height asymmetry was associated with decreased 10 m sprinting performance. The speed skating starting technique shares similarities with on-land starting techniques, where both require rapid SSC contractions of the lower limbs and demand a high level of reactive strength (Song et al., 2017). Therefore, an increase in VDJ jump height asymmetry might have adverse effects on speed skating starting performance. Furthermore, although the present study showed that increased TV and PF-L asymmetries in LDJ were associated with prolonged skating time, the results should be interpreted with caution due to the relatively long ground contact time in LDJ. Considering prolonged take-off time may not effectively reflect corresponding reactive strength, the association between the LDJ performance asymmetry and skating time may be more related to the concentric contraction performance of relevant muscles (Möck et al., 2023). It is recommended to prioritize the VDJ as the testing task when assessing SLDJ performance asymmetry, and that other directional SLDJ can be replaced by the SLSJ. Finally, it should be noted that, although the asymmetry magnitude of several variables in this study exceeded established injury threshold standards (10% or 15%) (Barber et al., 1990), we cannot definitively conclude that the subjects will face a higher risk of injury. Bishop (2021) suggest that the previously established injury thresholds of 10% or 15% lack a certain objectivity, due to the task-specific nature of IA. The sensitivity of IA varies significantly across different tasks and variables, which might explain the inconsistencies in the findings regarding the predictive value of IA for injury risk. Therefore, injury thresholds should not be generalized but rather defined based on the characteristics of specific tasks and variables.

This study has certain limitations. This study only contained adolescent male speed skaters, the group specificity restricted the sample size, and the results may only be applicable to the same demographic. Future research may increase the sample diversity and size to compare the relationship between lower limb asymmetry and athletic performance across different genders and age groups in speed skaters. Additionally, this study only assessed the lower limb asymmetry of athletes at a specific time point. It remains unclear how the lower limb IA of adolescent speed skaters changes longitudinally throughout the season. Future research could include longitudinal studies regarding the adolescent speed skaters.

## 5 Conclusion

This study indicate that, influenced by the asymmetric loads during corner, adolescent speed skaters exhibit differences in bilateral lower limb strength performance, which are task-specific. Most LSJ performance related variables consistently show a left-side dominance, and increases in corresponding IA are correlated with prolonged skating time. Additionally, increased VDJ jump height asymmetry may also have adverse effects on sprinting skating performance. Furthermore, considering the relatively sensitive asymmetry identification of LSJ and VDJ among adolescent speed skaters, it is recommended to monitor and evaluate the lower limb asymmetry in speed skaters based on aforementioned tasks.

## Data Availability

The raw data supporting the conclusions of this article will be made available by the authors, without undue reservation.
