# Supplementary material for: Does inter-limb asymmetry matter in adolescent speed skaters?
Source: Front Physiol. 2025 May 15;16:1498911. doi: 10.3389/fphys.2025.1498911 (PMC12119606; doi:10.3389/fphys.2025.1498911)
Supplement: Supplementary file 1 [file DataSheet1.docx]

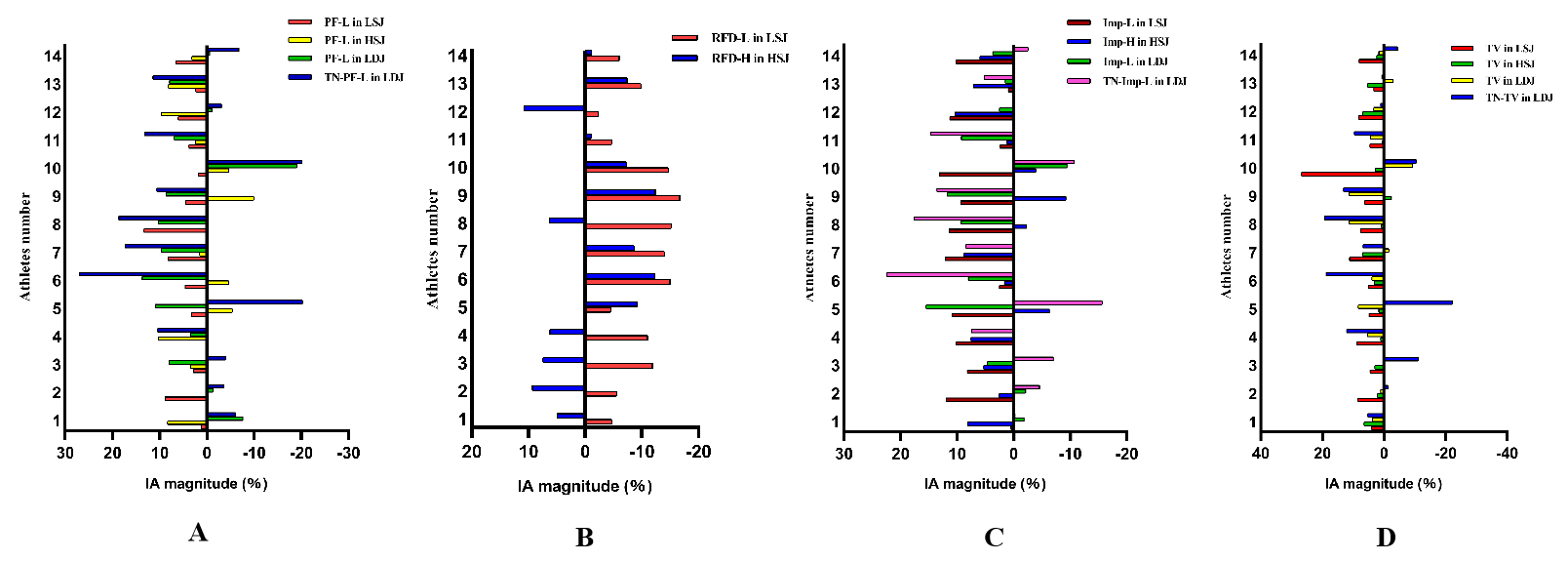


**Supplementary Figure S1.** Individual IA data in PF-L/H (A), RFD-L/H (B), Imp-L/H (C), TV-L/H (D). L, lateral; H, horizontal; PF, peak force; RFD, rate of force development; Imp, impulse; TV, take-off velocity.

**Supplementary Table S1.** Results of lower limb muscle mass and isokinetic test (Mean ± SD)

| Variables (n = 17) | L | R |
| --- | --- | --- |
| Muscle mass |  |  |
| Absolute lower limb muscle mass (kg) | 9.26 ± 1.49 | 9.30 ± 1.49 |
| Relative lower limb muscle mass (constant) | 0.15 ± 0.01 | 0.15 ± 0.01 |
| Isokinetic test |  |  |
| Relative knee extension PT at 60°/s (N∙m/kg)** | 2.20 ± 0.39 | 2.47 ± 0.43 |
| Relative knee flexion PT at 60°/s (N∙m/kg) | 2.00 ± 0.43 | 2.12 ± 0.51 |
| Relative knee extension PT at 180°/s (N∙m/kg) | 1.81 ± 0.30 | 1.91±0.43 |
| Relative knee flexion PT at 180°/s (N∙m/kg) | 1.82 ± 0.39 | 1.88 ± 0.40 |
| Relative knee extension PP at 60°/s (W/kg)** | 2.14 ± 0.36 | 2.47 ± 0.34 |
| Relative knee flexion PP at 60°/s (W/kg) | 1.81 ± 0.28 | 1.89 ± 0.27 |
| Relative knee extension PP at 180°/s (W/kg)* | 4.78 ± 0.79 | 5.33 ± 0.98 |
| Relative knee flexion PP at 180°/s (W/kg) | 4.38 ± 0.62 | 4.65 ± 0.76 |

L, left; R, right; PT, peak torque; PP, peak power; *, P < 0.05; **, P < 0.01.

**Supplementary Table S2.** Results of SLSJ test

| Variables  (n = 14) | | Vertical | | |  | Horizontal | | |  | Lateral | | |
| --- | --- | --- | --- | --- | --- | --- | --- | --- | --- | --- | --- | --- |
|  |  | Mean±SD | CV | ICC |  | Mean±SD | CV | ICC |  | Mean±SD | CV | ICC |
| Jump height (m)/ distance (constant) | L | 0.17±0.03 | 6.10 | 0.88(0.66-0.96) |  | 0.88±0.07 | 4.19 | 0.68(0.27-0.88) |  | 0.88±0.07 | 3.10 | 0.74(0.35-0.91) |
|  | R | 0.17±0.04 | 11.10 | 0.77(0.41-0.92) |  | 0.87±0.08 | 2.88 | 0.87(0.64-0.95) |  | 0.88±0.07 | 2.31 | 0.87(0.63-0.96) |
|  |  |  |  |  |  |  |  |  |  |  |  |  |
| Relative peak force -V (N/kg) b | L | 17.23±0.85 | 1.74 | 0.98(0.93-0.99) |  | 15.35±1.31 | 4.70 | 0.65(0.18-0.88 |  | 13.92±1.25 | 3.88 | 0.75(0.38-0.91) |
|  | R | 17.19±0.85 | 2.73 | 0.93(0.79-0.98) |  | 15.45±1.08 | 2.72 | 0.83(0.56-0.94) |  | 13.36±1.11 | 3.51 | 0.76(0.40-0.92) |
|  |  |  |  |  |  |  |  |  |  |  |  |  |
| RFD-V  (N/s) | L | 1114.63±278.98 | 8.11 | 0.80(0.47-0.93) |  | 465.62±138.14 | 15.31 | 0.73(0.25-0.91) |  | 283.44±86.17 | 15.46 | 0.70(0.27-0.89) |
|  | R | 1111.95±249.16 | 8.41 | 0.62(0.14-0.86) |  | 477.15±142.89 | 8.65 | 0.92(0.76-0.97) |  | 320.40±84.92 | 10.14 | 0.76(0.40-0.92) |
|  |  |  |  |  |  |  |  |  |  |  |  |  |
| Relative peak force -H/L (N/kg) b | L | — | — | — |  | 6.77±0.85 | 5.24 | 0.78(0.42-0.93) |  | 7.40±0.67 | 4.03 | 0.78(0.44-0.92) |
|  | R | — | — | — |  | 6.66±0.86 | 6.13 | 0.78(0.36-0.93) |  | 7.03±0.61 | 2.62 | 0.88(0.68-0.96) |
|  |  |  |  |  |  |  |  |  |  |  |  |  |
| RFD -H/L  (N/kg) b | L | — | — | — |  | 499.39±124.96 | 5.86 | 0.93(0.79-0.98) |  | 494.09±75.03 | 6.16 | 0.79(0.47-0.93) |
|  | R | — | — | — |  | 504.61±118.94 | 6.48 | 0.87(0.64-0.96) |  | 546.20±69.01 | 4.51 | 0.83(0.56-0.72) |
|  |  |  |  |  |  |  |  |  |  |  |  |  |
| Relative Imp-V (Ns/kg) | L | — | — | — |  | 1.32±0.21 | 11.40 | 0.63(0.15-0.86) |  | 1.06±0.26 | 11.02 | 0.82(0.53-0.94) |
|  | R | — | — | — |  | 1.26±0.18 | 7.28 | 0.80(0.47-0.93) |  | 1.03±0.23 | 8.30 | 0.88(0.67-0.96) |
|  |  |  |  |  |  |  |  |  |  |  |  |  |
| Relative Imp-H/L (Ns/kg) b | L | — | — | — |  | 2.55±0.22 | 5.56 | 0.86(0.58-0.96) |  | 2.67±0.23 | 2.52 | 0.98(0.94-0.99) |
|  | R | — | — | — |  | 2.48±0.23 | 3.84 | 0.94(0.76-0.98) |  | 2.45±0.24 | 2.83 | 0.98(0.93-0.99) |
|  |  |  |  |  |  |  |  |  |  |  |  |  |
| Take-off velocity (m/s) a, b | L | — | — | — |  | 2.84±0.17 | 3.43 | 0.56(0.09-0.83) |  | 2.81±0.20 | 1.90 | 0.88(0.66-0.96) |
|  | R | — | — | — |  | 2.76±0.18 | 3.41 | 0.58(0.12-0.84) |  | 2.59±0.30 | 2.66 | 0.93(0.81-0.98) |

V, vertical; H, horizontal; L, lateral; Imp, impulse; RFD, rate of force development; Imp, impulse; CV, coefficient of variation; ICC, intra-class correlation coefficient; a, significant bilateral differences in horizontal jump (P < 0.05); b, significant bilateral differences in lateral jump (P < 0.05).

**Supplementary Table S3.** Results of SLDJ test

| Variables  (n = 17) | | Vertical | | |  | Horizontal | | |  | Lateral | | |
| --- | --- | --- | --- | --- | --- | --- | --- | --- | --- | --- | --- | --- |
|  |  | Mean±SD | CV | ICC |  | Mean±SD | CV | ICC |  | Mean±SD | CV | ICC |
| Jump height (m)/ distance (constant) | L | 0.16±0.05 | 13.47 | 0.75(0.43-0.90) |  | 0.86±0.06 | 2.59 | 0.81(0.56-0.72) |  | 0.89±0.10 | 2.12 | 0.95(0.81-0.98) |
|  | R | 0.18±0.04 | 10.03 | 0.77(0.47-0.91) |  | 0.88±0.06 | 2.57 | 0.78(0.49-0.92) |  | 0.93±0.07 | 1.83 | 0.91(0.76-0.97) |
| Relative peak force -V (N/kg) b | L | 21.55±2.27 | 3.87 | 0.81(0.54-0.93) |  | 18.32±2.83 | 4.48 | 0.95(0.86-0.98) |  | 16.62±1.60 | 4.07 | 0.93(0.81-0.97) |
|  | R | 21.53±3.23 | 4.01 | 0.90(0.76-0.96) |  | 18.54±2.79 | 3.66 | 0.94(0.85-0.98) |  | 15.95±1.56 | 2.90 | 0.97(0.91-0.99) |
| Relative peak force -H/L (N/kg) | L | — | — | — |  | 6.53±1.07 | 6.41 | 0.88(0.64-0.96) |  | 7.86±1.06 | 5.43 | 0.93(0.82-0.97) |
|  | R | — | — | — |  | 6.26±1.28 | 6.05 | 0.93(0.81-0.98) |  | 7.53±1.00 | 4.67 | 0.94(0.84-0.98) |
| Relative Imp-V  (Ns/kg) | L | — | — | — |  | 1.31±0.23 | 13.84 | 0.64(0.22-0.86) |  | 1.27±0.25 | 12.40 | 0.56(0.15-0.81) |
|  | R | — | — | — |  | 1.42±0.20 | 6.56 | 0.83(0.59-0.93) |  | 1.27±0.27 | 6.46 | 0.92(0.78-0.97) |
| Relative Imp-H/L (Ns/kg) b | L | — | — | — |  | 1.68±0.21 | 6.55 | 0.82(0.57-0.93) |  | 2.67±0.26 | 3.73 | 0.95(0.87-0.98) |
|  | R | — | — | — |  | 1.62±0.25 | 7.72 | 0.86(0.48-0.96) |  | 2.54±0.25 | 3.30 | 0.97(0.91-0.99) |
| Take-off velocity (m/s) b | L | — | — | — |  | 2.69±0.25 | 3.86 | 0.77(0.47-0.91) |  | 2.85±0.24 | 1.97 | 0.90(0.73-0.96) |
|  | R | — | — | — |  | 2.66±0.24 | 3.32 | 0.78(0.41-0.92) |  | 2.74±0.24 | 2.43 | 0.89(0.74-0.96) |

V, vertical; H, horizontal; L, lateral; Imp, impulse; Imp, impulse; CV, coefficient of variation; ICC, intra-class correlation coefficient; b, significant bilateral differences in lateral jump (P < 0.05).

**Supplementary Table S4.** Results of time-normalized SLDJ test

| Variables  (n = 17) | | Vertical | | |  | Horizontal | | |  | Lateral | | |
| --- | --- | --- | --- | --- | --- | --- | --- | --- | --- | --- | --- | --- |
|  |  | Mean±SD | CV | ICC |  | Mean±SD | CV | ICC |  | Mean±SD | CV | ICC |
| TN-Jump height (m)/ distance (constant) | L | 0.37±0.12 | 13.45 | 0.81(0.55-0.93) |  | 1.61±0.30 | 6.40 | 0.83(0.59-0.94) |  | 1.41±0.33 | 7.82 | 0.80(0.54-0.92) |
|  | R | 0.38±0.12 | 10.64 | 0.87(0.69-0.95) |  | 1.71±0.28 | 5.63 | 0.82(0.57-0.72) |  | 1.44±0.26 | 6.50 | 0.82(0.54-0.93) |
| TN-Relative peak force -V (N/kg) | L | 49.55±15.40 | 9.63 | 0.87(0.68-0.95) |  | 35.27±12.09 | 8.63 | 0.92(0.80-0.97) |  | 26.54±7.49 | 10.56 | 0.85(0.64-0.94) |
|  | R | 47.47±19.83 | 9.33 | 0.95(0.86-0.98) |  | 36.74±11.31 | 8.50 | 0.86(0.66-0.95) |  | 25.03±6.66 | 8.62 | 0.93(0.79-0.97) |
| TN-Relative peak force -H/L (N/kg) | L | — | — | — |  | 12.46±3.66 | 9.50 | 0.90(0.70-0.96) |  | 12.49±3.51 | 9.27 | 0.87(0.69-0.95) |
|  | R | — | — | — |  | 12.20±3.27 | 8.10 | 0.93(0.82-0.98) |  | 11.70±2.62 | 8.82 | 0.90(0.72-0.96) |
| TN-Relative Imp-V  (Ns/kg) | L | — | — | — |  | 2.46±0.66 | 13.46 | 0.73(0.39-0.89) |  | 2.01±0.58 | 12.31 | 0.79(0.52-0.92) |
|  | R | — | — | — |  | 2.76±0.66 | 8.39 | 0.78(0.50-0.91) |  | 1.98±0.63 | 8.42 | 0.92(0.80-0.97) |
| TN-Relative Imp-H/L (Ns/kg) b | L | — | — | — |  | 3.16±0.74 | 10.22 | 0.77(0.48-0.91) |  | 4.22±0.94 | 7.55 | 0.88(0.70-0.95) |
|  | R | — | — | — |  | 3.11±0.56 | 7.37 | 0.78(0.44-0.92) |  | 3.93±0.71 | 7.53 | 0.91(0.74-0.97) |
| TN-Take-off velocity (m/s) | L | — | — | — |  | 5.07±1.10 | 7.56 | 0.85(0.63-0.94) |  | 4.50±0.98 | 7.34 | 0.82(0.57-0.93) |
|  | R | — | — | — |  | 5.15±0.83 | 6.20 | 0.82(0.56-0.93) |  | 4.25±0.81 | 7.15 | 0.80(0.51-0.92) |

V, vertical; H, horizontal; L, lateral; Imp, impulse; Imp, impulse; TN, time-normalized; CV, coefficient of variation; ICC, intra-class correlation coefficient; b, significant bilateral differences in lateral jump (P < 0.05).

**Supplementary Table S5.** Kappa coefficient between different angular velocity in knee isokinetic tests

| Variables  (n = 17) | 60°/s VS 180°/s | | | | |
| --- | --- | --- | --- | --- | --- |
|  | Extension | Description |  | Flexion | Description |
| Peak torque | 0.43 | Moderate |  | 0.33 | Fair |
| Peak power | 0.14 | Trivial |  | 0.46 | Moderate |

**Supplementary Table S6.** Kappa coefficient between different direction SLSJ

| Variables  (n = 14) | VSJ VS HSJ | Description |  | VSJ VS LSJ | Description |  | HSJ VS LSJ | Description |
| --- | --- | --- | --- | --- | --- | --- | --- | --- |
| Jump height/distance | -0.04 | Trivial |  | -0.17 | Trivial |  | 0.55 | Moderate |
| Relative peak force -V | -0.08 | Trivial |  | 0.65 | Substantial |  | -0.08 | Trivial |
| RFD  -V | 0.21 | Fair |  | 0.36 | Fair |  | -0.14 | Trivial |
| Relative peak force - H/L | — | — |  | — | — |  | 0* | CC |
| RFD  -H/L | — | — |  | — | — |  | 0* | CC |
| Relative  Imp-V | — | — |  | — | — |  | -0.29 | Trivial |
| Relative  Imp-H/L | — | — |  | — | — |  | 0* | CC |
| Take-off  velocity | — | — |  | — | — |  | 0* | CC |

VSJ, vertical single-leg squat jump; HSJ, horizontal single-leg squat jump; LSJ, lateral single-leg squat jump; CC, cannot be calculated.

**Supplementary Table S7.** Kappa coefficient between different direction SLDJ

| Variables  (n = 17) | VDJ VS HDJ | Description |  | VDJ VS LDJ | Description |  | HDJ VS LDJ | Description |
| --- | --- | --- | --- | --- | --- | --- | --- | --- |
| **General** |  |  |  |  |  |  |  |  |
| Jump height/distance | 0.25 | Fair |  | 0.25 | Fair |  | -0.13 | Trivial |
| Relative peak force -V | 0.17 | Trivial |  | -0.12 | Trivial |  | 0.44 | Moderate |
| Relative peak force - H/L | — | — |  | — | — |  | -0.13 | Trivial |
| Relative  Imp-V | — | — |  | — | — |  | -0.04 | Trivial |
| Relative  Imp-H/L | — | — |  | — | — |  | -0.02 | Trivial |
| Take-off  velocity | — | — |  | — | — |  | 0.27 | Fair |
| **Time**  **-Normalized** |  |  |  |  |  |  |  |  |
| TN-Jump height/distance | 0.27 | Fair |  | 0.17 | Trivial |  | -0.07 | Trivial |
| TN-Relative peak force -V | 0.19 | Trivial |  | 0.16 | Trivial |  | 0.20 | Trivial |
| TN-Relative peak force - H/L | — | — |  | — | — |  | 0.17 | Trivial |
| TN-Relative  Imp-V | — | — |  | — | — |  | -0.08 | Trivial |
| TN-Relative  Imp-H/L | — | — |  | — | — |  | 0.38 | Fair |
| TN-Take-off  velocity | — | — |  | — | — |  | 0.31 | Fair |

VDJ, vertical single-leg drop jump; HDJ, horizontal single-leg drop jump; LDJ, lateral single-leg drop jump.

**Supplementary Table S8.** Kappa coefficient between SLSJ and SLDJ

| Variables  (n = 14) | VSJ VS VDJ | Description |  | HSJ VS HDJ | Description |  | LSJ VS LDJ | Description |
| --- | --- | --- | --- | --- | --- | --- | --- | --- |
| **General** |  |  |  |  |  |  |  |  |
| Jump height/distance | 0.46 | Moderate |  | -0.15 | Trivial |  | -0.08 | Trivial |
| Relative peak force -V | 0.29 | Fair |  | 0.29 | Fair |  | 0.19 | Trivial |
| Relative peak force - H/L | — | — |  | 0.22 | Fair |  | 0* | CC |
| Relative  Imp-V | — | — |  | 0.31 | Fair |  | 0.14 | Trivial |
| Relative  Imp-H/L | — | — |  | 0.30 | Fair |  | 0* | CC |
| Take-off  velocity | — | — |  | -0.13 | Trivial |  | 0* | CC |
| **Normalized** |  |  |  |  |  |  |  |  |
| TN-Jump height/distance | 0.46 | Moderate |  | 0.21 | Fair |  | -0.12 | Trivial |
| TN-Relative peak force -V | 0.09 | Trivial |  | 0.57 | Moderate |  | 0.30 | Fair |
| TN-Relative peak force - H/L | — | — |  | 0 | Trivial |  | 0* | CC |
| TN-Relative  Imp-V | — | — |  | 0.34 | Fair |  | 0 | Trivial |
| TN-Relative  Imp-H/L | — | — |  | 0.09 | Trivial |  | 0* | CC |
| TN-Take-off  velocity | — | — |  | 0.11 | Trivial |  | 0* | CC |

**Supplementary Table S9.** Asymmetry magnitude of muscle mass and isokinetic tests

| Variables（n=17） | IA |
| --- | --- |
| **Muscle mass** |  |
| Lower limb muscle mass | 0.92±0.64 |
| **Isokinetic test** |  |
| Relative knee extension PT at 60°/s | 12.87±9.74 |
| Relative knee flexion PT at 60°/s | 11.31±8.19 |
| Relative knee extension PT at 180°/s | 14.05±9.82 |
| Relative knee extension PT at 180°/s | 13.38±9.34 |
| Relative knee extension PP at 60°/s | 12.98±11.39 |
| Relative knee extension PP at 60°/s | 6.33±4.01 |
| Relative knee extension PP at 180°/s | 15.52±11.35 |
| Relative knee extension PP at 180°/s | 9.99±7.32 |

**Supplementary Table S10.** Asymmetry magnitude of SLSJ and SLDJ tests

| Variables | General (Mean±SD) | | |  | Time-normalized (Mean±SD) | | |
| --- | --- | --- | --- | --- | --- | --- | --- |
|  | Vertical | Horizontal | Lateral |  | Vertical | Horizontal | Lateral |
| SLSJ (n=14) |  |  |  |  |  |  |  |
| Jump height/distance | 11.95±10.52 | 5.20±3.41 | 2.42±1.72 |  |  |  |  |
| Relative peak force -V | 3.34±2.75 | 3.78±2.01 | 4.57±4.94 |  |  |  |  |
| RFD-V | 13.94±9.82 | 12.46±8.81 | 16.73±9.11 |  |  |  |  |
| Relative peak force -H/L |  | 5.18±3.56 | 4.82±3.55 |  |  |  |  |
| RFD -H/L |  | 7.52±3.47 | 9.77±4.94 |  |  |  |  |
| Relative Imp-V |  | 10.54±5.78 | 11.60±7.00 |  |  |  |  |
| Relative Imp-H/L |  | 5.76±3.00 | 8.21±4.54 |  |  |  |  |
| Take-off velocity |  | 3.27±2.24 | 8.00±5.88 |  |  |  |  |
| SLDJ (n=17) |  |  |  |  |  |  |  |
| Jump height/distance | 13.17±10.55 | 5.03±3.83 | 6.50±5.67 |  | 18.07±14.10 | 11.60±9.52 | 10.59±9.04 |
| Relative peak force -V | 7.13±6.50 | 6.80±6.62 | 4.91±3.85 |  | 19.94±13.84 | 15.49±12.76 | 9.89±7.81 |
| Relative peak force -H/L |  | 8.04±5.03 | 7.64±4.99 |  |  | 9.23±8.31 | 12.55±7.11 |
| Relative Imp-V |  | 14.84±10.69 | 15.08±9.23 |  |  | 20.35±15.26 | 17.77±12.28 |
| Relative Imp-H/L |  | 9.50±6.15 | 6.45±4.86 |  |  | 9.22±6.83 | 10.56±6.98 |
| Take-off velocity |  | 4.31±2.87 | 5.71±4.19 |  |  | 9.84±7.96 | 10.87±7.15 |
